# Inhibition Effect of Chloroquine and Integrin-Linked Kinase Knockdown on Translation in Melanoma Cells

**DOI:** 10.3390/ijms22073682

**Published:** 2021-04-01

**Authors:** Dorota Gil, Piotr Laidler, Marta Zarzycka, Joanna Dulińska-Litewka

**Affiliations:** Chair of Medical Biochemistry, Jagiellonian University Medical College, ul. Kopernika 7, 31-034 Kraków, Poland; piotr.laidler@uj.edu.pl (P.L.); marta.zarzycka@uj.edu.pl (M.Z.); joanna.dulinska-litewka@uj.edu.pl (J.D.-L.)

**Keywords:** ILK, chloroquine, mTOR, AMPK, melanoma, eIF4E, eIF2α, LC3

## Abstract

The twofold role of autophagy in cancer is often the therapeutic target. Numerous regulatory pathways are shared between autophagy and other molecular processes needed in tumorigenesis, such as translation or survival signaling. Thus, we have assumed that ILK knockdown should promote autophagy, and used together with chloroquine, an autophagy inhibitor, it could generate a better anticancer effect by dysregulation of common signaling pathways. Expression at the protein level was analyzed using Western Blot; siRNA transfection was done for ILK. Analysis of cell signaling pathways was monitored with phospho-specific antibodies. Melanoma cell proliferation was assessed with the crystal violet test, and migration was evaluated by scratch wound healing assays. Autophagy was monitored by the accumulation of its marker, LC3-II. Our data show that ILK knockdown by siRNA suppresses melanoma cell growth by inducing autophagy through AMPK activation, and simultaneously initiates apoptosis. We demonstrated that combinatorial treatment of melanoma cells with CQ and siILK has a stronger antitumor effect than monotherapy with either of these. It generates the synergistic antitumor effects by the decrease of translation of both global and oncogenic proteins synthesis. In our work, we point to the crosstalk between translation and autophagy regulation.

## 1. Introduction

The key role played by autophagy in cancer is undisputed, but whether it is tumor-suppressive or tumor-promoting remains a controversial matter. Autophagy can suppress the initiation and development of a tumor, but on the other hand, it is employed by the tumor for adaptation to stress or starvation, and in response to cancer therapies [1,2]. Autophagy is essential for survival and development because it is a homeostatic cellular recycling pathway for degrading damaged or unnecessary proteins or even whole organelles. Low levels of autophagy maintain cell homeostasis by eliminating damaged molecules or, in the case of starvation, by supplying amino acids from degraded proteins [3]. In cancer biology, autophagy plays a dual role in cell survival and cell death. On the one hand, autophagy inhibits the growth and development of cancer, and on the other, it allows it to survive during chemotherapy [1,2,3,4,5,6]. Cancer cells are more autophagy-dependent than normal cells, thus, targeting autophagy is a therapeutic strategy for cancer therapy [4,5,6]. In melanoma, autophagy plays a dual role in cell survival and cell death. It has been shown that autophagy by itself suppressed melanogenesis and in addition it was reported that inhibition of autophagy may play a tumor-suppressing role. In the early stage of melanoma and other cancers, autophagy inhibits the tumor process, whereas in the advanced stage it seems to be an adaptive mechanism and acts as a tumor survival mechanism or even a tumor promoter [2,7].

Autophagy is regulated by mTOR, AMPK, and extracellular signals regulating kinase ERK. mTOR is a major cell-growth regulator controlling the expression of cellular proteins by integration signals from growth factors and nutrients. Under nutrition-rich conditions or in the presence of growth factors signals, mTOR inhibits autophagy. The AMP‑activated protein kinase (AMPK) promotes autophagy in response to the low energy of glucose deprivation. AMPK is also known to stimulate autophagy by inhibition of mTORC1 in a TSC-dependent manner or by direct phosphorylation of RAPTOR, an essential component mTORC1 complex. ERK is a downstream effector of MAPK and BRAF signaling. Common BRAF^V600E^ mutation in melanoma results in hyper-activation of the MAPK/ERK pathways, which correlates with MNKs-associated increase of phosphorylation of eIF4E, and favors tumorigenesis. The use of BRAF inhibitors develops a stress response—autophagy as a mechanism of drug resistance in melanoma cells. Thus, induction of disturbances in the autophagy process seems to be a promising melanoma treatment strategy in either a monotherapy or a combination therapy [8]. Chloroquine, which is a well-known antimalarial drug, increases the pH of lysosomes and effectively inhibits fusion of autophagosomes and lysosomes, thus interfering with protein degradation [9]. It is a well-accepted view that chloroquine (CQ) efficiently inhibits autophagy, and also shows promise for cancer treatment. The anti-cancer effect of chloroquine has been observed both in vitro and in vivo, in monotherapy as well as in combination with conventional anti-cancer treatments according to a new clinical study published in ecancermedicalscience [10]. Apart from autophagy inhibition, the pharmacological anti-cancer profile of chloroquine appears to also involve other mechanisms such as inhibition of proliferation, induction of apoptosis, or influencing tumor vasculature [11,12,13,14].

The multi-faceted anti-cancer action of CQ makes this drug attractive for cancer therapy. Recent reports show that combination treatment with autophagy inducers and inhibitors in cancer cells enhance the effect of anti-cancer therapies [8,15]. The sensitivity of melanoma cells to chloroquine was shown as independent of the BRAF mutation status [16]. For this reason, we decided to use CQ, which has been reported to exert anticancer effects on melanoma and other tumors [11,12,16], in combination with silencing of ILK (integrin-linked kinase) to strengthen siILK-caused inhibition of pro-survival signaling with CQ-driven autophagy inhibition. ILK takes part in various cell functions, such as cell-extracellular matrix interactions, cell cycle, apoptosis, cell proliferation, cell motility, and induction of epithelial-mesenchymal transition (EMT), which are related to the interacting partners of ILK and downstream signaling pathways [17,18]. We demonstrated that inhibition of ILK expression and activity using siRNA has antitumor effects in melanoma and bladder cancer [19,20,21]. ILK is a critical regulator of the cancer-survival pathway, including PI3K/AKT, glycogen synthase kinase 3β (GSK3β), nuclear factor-kappaβ (NF-κβ), mTOR, or Snail1/E-cadherin/N-cadherin [20,22,23]. Since PI3K/Akt/mTOR axis activation results in autophagy inhibition [24], we assume that the knockdown of ILK promotes autophagy.

We have previously shown that ILK silencing has anti-tumor implications for both melanoma and bladder cancer [19,20]. The present study is aimed at exploring the effects of CQ alone or in combination with silencing of ILK on melanoma cells and pathways associated with these treatments, as well as identifying potential molecular targets for melanoma treatment.

## 2. Results

### 2.1. Silencing of ILK and Chloroquine Inhibits the Survival of Melanoma Cells

**Figure 1:**
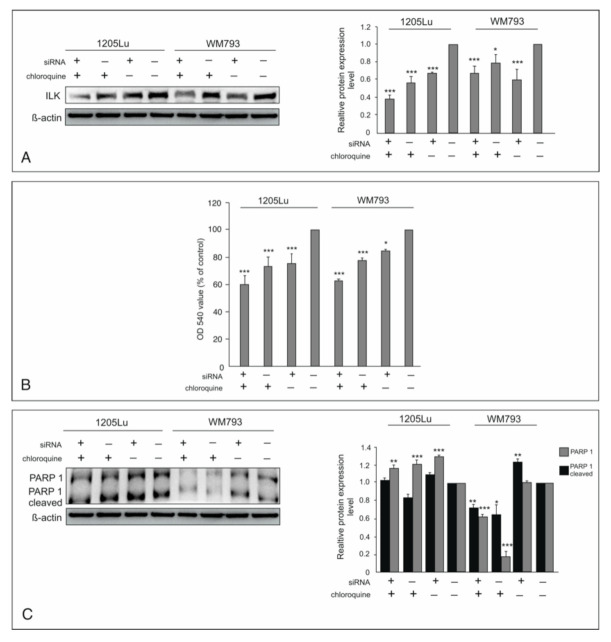
The inhibitory effect of ILK knockdown and CQ use on the survival of melanoma cells. “+” refers to siRNA ILK or chloroquine; “−” refers to silencer negative control cells. All results are presented as % of control; asterisk (*) indicates a significant difference: * *p* < 0.05, ** *p* < 0.01, *** *p* < 0.001. (**A**)—the expression of ILK after silencing detected by Western blotting in the presence or absence of CQ. (**C**)—Effect of ILK knock-down and CQ use on cleaved PARP-1. Representative blots are displayed and quantitative representation of data after densitometry (mean ± SD) of three independent experiments on histograms. Β-actin was used as a protein loading control. (**B**)—Cell proliferation was assessed with the crystal violet test. Values are expressed as mean ± standard deviation in 6 wells in three independent experiments.

Elevated ILK expression and activity contributes to increased proliferation and invasive potential of melanoma cells [19,25]. ILK knockdown reduced cell viability in both cell lines by about 25% in comparison to control cells (Figure 1A,B). Chloroquine also inhibits the growth and survival of melanoma cells to a similar extent as ILK silencing (Figure 1A,B). Simultaneous use of siRNA for ILK and chloroquine markedly affected cell growth and showed a 40% reduction in cell viability (Figure 1B). The increased level of cleaved Poly (ADP-ribose) polymerase (PARP1) indicates that apoptosis may contribute to cell death after silencing of ILK at least in WM793 and early-stage melanoma cells (Figure 1C). PARP1 plays a role in DNA repair; it is activated by binding to DNA ends or strand breaks. The cleavage of PARP1 catalyzed by caspase-3 causes inactivation of PARP1 [26].

### 2.2. Knockdown of ILK-Induced Autophagy

**Figure 2:**
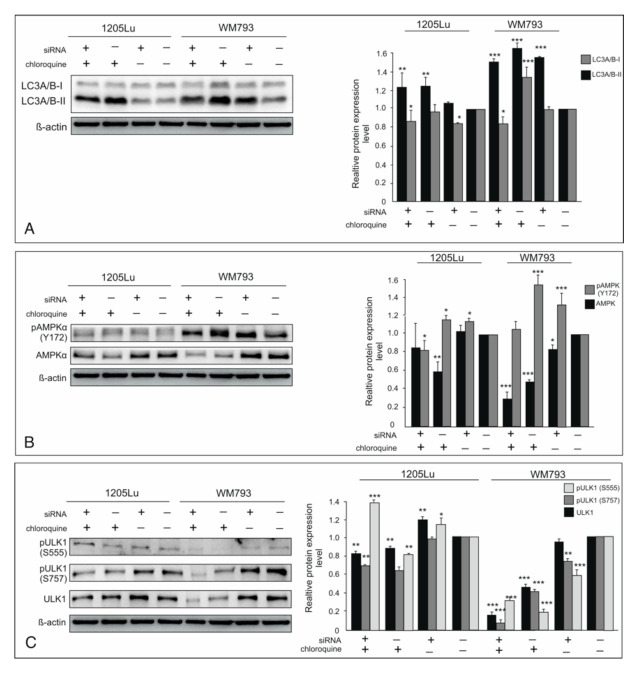
Effect of ILK knockdown on autophagy induction. “+” refers to siRNA ILK or chloroquine; “−” refers to silencer negative control cells. The expression of (**A**)—LC3-II, both phosphorylated and total (**B**)—AMPK, (**C**)—ULK1 were determined by Western blotting. Representative blots are displayed. The histograms are a quantitative representation of data after densitometry (mean ± SD) of three independent experiments. β-actin was used as a protein loading control. Asterisks indicate significant differences from control cells. Values are denoted as * *p* < 0.05, ** *p* < 0.01, and *** *p* < 0.001.

During autophagy, LC3 (microtubule-associated protein light chain 3) is cleaved off by Atg4 (cysteine protease) generating LC3-I, which is conjugated to phosphatidylethanolamine to form LC3-II. While LC3-I stays in the cytoplasm, LC3-II binds to both the outer and the inner membranes of the autophagosome [27]. We examined if silencing of ILK by siRNA affects LC3-II level used as a marker of autophagy progression. Both studied melanoma cells exhibit a high level of basal autophagy but respond differently to the knockdown of ILK. After silencing of ILK, WM793 cells accumulated LC3-II similarly to the CQ treatment (Figure 2A). In 1205Lu, we observed an uptick in LC3-II levels not comparable with the CQ effect, and a decrease in LC3-I form (Figure 2A). The knockdown of ILK seems to activate autophagy more in the early stages of melanoma development than in metastatic cells.

### 2.3. ILK Inhibits AMPK-Dependent Autophagy

Autophagy is promoted by AMP-activated protein kinase (AMPK) as an energy receptor. Activation of AMPK by phosphorylation at Thr172 in catalytic subunit α promotes autophagy initiation as the major regulator. Phosphorylation of the AMPK α-subunit at Thr172 is controlled by upstream kinases: liver kinase B1 (LKB1) and calcium/calmodulin-dependent protein kinase-β (CaMKKβ). Furthermore, the Akt/GSK signaling pathway is involved in the regulation of AMPK activity. GSK3 binds with AMPKβ prior to phosphorylation of AMPKα, which facilitates AMPKα binding to phosphatases, and therefore inactivates AMPK. Akt signaling normally inhibits GSK3 activity, promotes GSK3 phosphorylation at S9, and in effect leads to inhibition of AMPK [28]. Phosphorylation of AMPK was also observed in chloroquine treatment in myotubes [29]. We showed earlier that in cancer cells, ILK phosphorylates and activates Akt at Ser 473. ILK also directly phosphorylates GSK-3β at Ser 9, thus inactivating it [19,20]. siRNA-mediated ILK knockdown resulted in the activation of AMPK by its increased phosphorylation at Thr172, which was significant in WM793, and not as substantial in 1205Lu (Figure 2B). The increase of phosphorylation of AMPK is also visible after CQ treatment alone as well as in combination with siILK (Figure 2B).

### 2.4. ILK Knockdown is Sufficient to Induce Autophagy Through Regulation of ULK1 Activity

ULK1 is a critical mammalian autophagy-initiating Ser/Thr kinase necessary for ULK1/Atg13/FIP200 complex formation. The activated ULK complex localizes to the phagophore and activates the PI3K complex responsible for nucleation of the next phase of autophagy processes. The ULK complex formation in a phosphorylation-dependent manner is suppressed by mTORC1. The active mTORC1 phosphorylates S757 and prevents ULK1 interaction with the rest of the complex [30]. When mTORC1 is inhibited, dephosphorylated ULK1 at S757 forms an active complex with Atg13 and FIP200. ULK1 can also be directly phosphorylated by AMPK at a site distinct from the mTORC1 one. The AMPK-phosphorylated ULK1 at S317, S777, and S555 is active and able to initiate autophagy. ULK1 can interact with and be activated by AMPK only when mTORC1 phosphorylation of S757 is decreased [31]. As shown in Figure 2C, ILK knockdown decreased ULK1 phosphorylation at S757, which probably enabled its interaction with AMPK in the melanoma cell line WM793. In the more advanced melanoma cell line 1205Lu, we observe a markedly higher increase of ULK expression after silencing of ILK. Moreover, activation of ULK1 by AMPK is visible as increased phosphorylation on S555 in comparison to control cells (Figure 2C). These data indicate that ILK likely regulates autophagy through the coordinate activity of mTORC1 and AMPK.

### 2.5. mTOR Activity Regulated by ILK and Chloroquine

**Figure 3:**
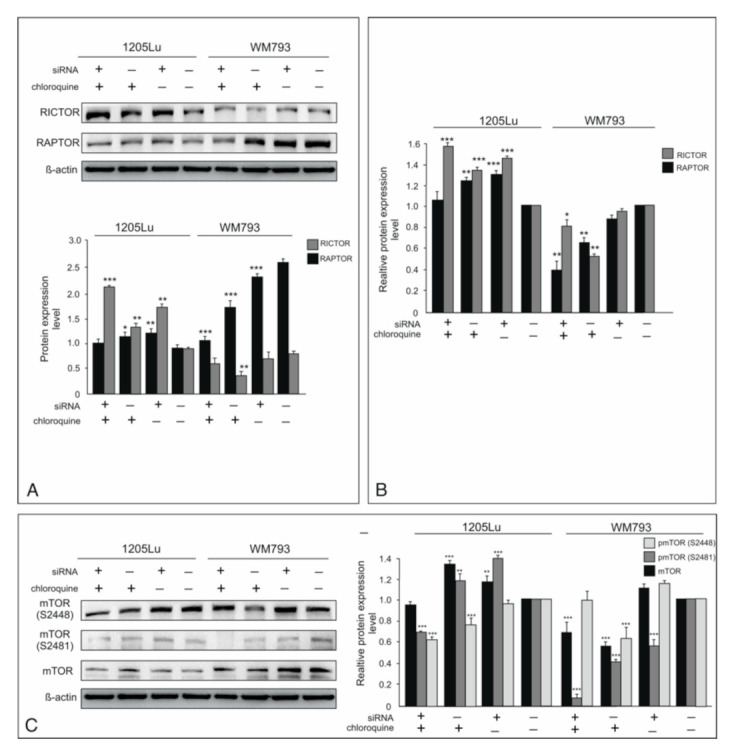
Effect of ILK knockdown and CQ use on mTOR activity. “+” refers to siRNA ILK or chloroquine; “−” refers to silencer negative control cells. (**A**)—Expression RICTOR and RAPTOR. Representative blots are displayed. The histogram presented protein level expression. (**B**)—The histogram presented relative protein expression level after densitometry (mean ± SD) of three independent experiments. β-actin was used as a protein loading control. Asterisks indicate significant differences from control cells. Values are denoted as * *p* < 0.05, ** *p* < 0.01, and *** *p* < 0.001. (**C**)—the expression of both phosphorylated and total mTOR. Representative blots are displayed. The histograms are a quantitative representation of data after densitometry (mean ± SD) of three independent experiments. β-actin was used as a protein loading control. Asterisks indicate significant differences from control cells. Values are denoted as * *p* < 0.05, ** *p* < 0.01, and *** *p* < 0.001.

mTOR, together with its pathway components, is known as a negative regulator of autophagy. mTOR forms two multi-protein complexes because mTOR kinase can be associated with various regulatory proteins. mTORC1 is defined by RAPTOR (regulatory-associated protein of mTOR), while mTORC2 is determined by RICTOR (rapamycin-insensitive companion of mTOR). mTORC1 is activated by the Akt pathway and integrates signals from outside the cell with the translational machinery. mTORC2 is involved in cell cycle progression and cell survival by phosphorylation of S473 on Akt [24]. We found that the WM793 cell line has more RAPTOR than RICTOR protein, but the exact opposite is the case with the 1205 Lu cell line (Figure 3A). The expression of these proteins drops, especially after CQ treatment alone or in combination with siRNA for ILK, but only in the WM793 cell line. In 1205 Lu cells, we observed a marked increase in expression in RICTOR, in particular after silencing of ILK or chloroquine treatment, as well as simultaneous use of both (Figure 3B). The decreased phosphorylation status of S2448 specific for mTORC1 was visible in 1205Lu cells after their treatment with chloroquine alone or in combination with siRNA for ILK, but not for the latter used alone. Increased mTOR kinase protein level is accompanied by increased S2481 phosphorylation specific for mTORC2 in this line after silencing of ILK or CQ treatment, but when both were used simultaneously, the activity of both mTORC2 and mTORC1 decreased. On the contrary, a marked decrease of S2481 phosphorylation (mTORC2) was noted in WM793 cells after ILK silencing, chloroquine treatment, or simultaneous use of both. Additionally, we observed a slight increase of the mTOR protein level, which co-occurred with a S2448 phosphorylation (mTORC1) increase in cells with knocked down ILK (Figure 3C). The surprising results of the cell response may stem from the a priori different expression of RICTOR and RAPTOR in the tested lines.

Important targets of mTORC1 in translational control are 4E-BP1 (eukaryotic translation initiation factor 4E-binding protein) and p70S6K (ribosomal protein p70S6 kinase) [24]. The basal expression levels of p70S6K decreased significantly after silencing of ILK and drastically after CQ treatment alone or in combination with siRNA for ILK in both studied cell lines. This effect was not consistent with the phosphorylation status. Phosphorylation of Thr389 induces a conformational change that exposes Thr229 in the activation loop of p70S6K and allows its phosphorylation by PDK1, resulting in p70S6K activation [32].

**Figure 4:**
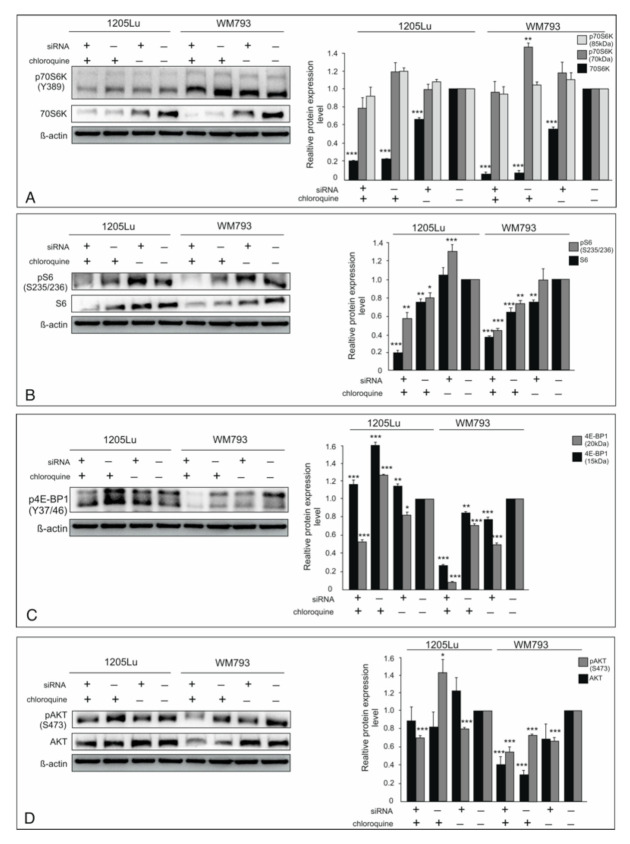
The effect of ILK silencing and CQ use on downstream of the mTOR signaling pathway. “+” refers to siRNA ILK or chloroquine; “−” refers to silencer negative control cells. Protein expression, both phosphorylated and total, was assessed by Western blotting. Representative blots are displayed. The histograms are a quantitative representation of data after densitometry (mean ± SD) of three independent experiments. β-actin was used as a protein loading control. Asterisks indicate significant differences from control cells. Values are denoted as * *p* < 0.05, ** *p* < 0.01, and *** *p* < 0.001. (**A**)—ribosomal protein p70S6 kinase, (**B**)—ribosomal protein S6, (**C**)—eukaryotic translation initiation factor 4E-binding protein (4E-BP-1)-phosphorylated form, (**D**)—Akt expression.

The phosphorylation on Thr 389 p70S6 decreased insignificantly only when ILK and CQ were used simultaneously. When either CQ or siRNA for ILK were used independently, the p70S6K activity monitored as its phosphorylation on Y389 has even increased (Figure 4A). The unexpectedly higher phosphorylation in relation to the reduced amount of protein results from the fact that p70SK is a protein not exclusively phosphorylated by mTOR, but also phosphoinositide 3-kinase (PI3K) signaling, mitogen-activated protein kinase (MAPK) signaling, and even p70S6K autophosphorylation [32].

In the case of ribosomal protein S6, a substrate for p70S6K, we detected that the decrease in protein expression was accompanied by a decrease in phosphorylation in the WM793 and 1205 Lu cells treated with CQ alone or in combination with siRNA for ILK. We noticed increased expression as well as the phosphorylation level of rpS6 in 1205 Lu after knockdown of ILK (Figure 4B). Another important substrate of mTORC1 is 4E-BP1, a small translational repressor, which after phosphorylation by mTORC1 releases eIF4E. Our results showed increased repression activity of 4EBP1 in response to ILK silencing or CQ treatment only in WM793, and their simultaneous use was more effective when compared to monotherapy in both tested cell lines (Figure 4C). CQ use in monotherapy in 1205Lu cells caused a very significant increase of the hyper-phosphorylated form 4EBP1, which weakens its interaction with eIF4E (Figure 4C).

The main known mTORC2 activity is the phosphorylation and activation of Akt, but it is not the only factor activating this critical cell fate upstream kinase [33]. We showed that kinase ILK can also activate the Akt [19,20]. Therefore, it comes as no surprise that the activation of Akt decreases upon ILK silencing alone or in combination with CQ. Reduction of basal expression of Akt resulted in downregulation of pAkt in WM793 treated with CQ alone or in combination with siRNA for ILK, and the decrease of Akt activity was more prominent in the case of simultaneous use of both. However, treatment with CQ alone resulted in an increased Akt activity in 1205Lu, a metastatic melanoma cell line. A relatively higher level of phosphorylation of Akt in 1205Lu in the presence of CQ suggests a higher activity of mTORC2 in these cells (Figure 4D).

### 2.6. Targeting the Translational Machinery by Silencing ILK, CQ, and Combination Treatment

**Figure 5:**
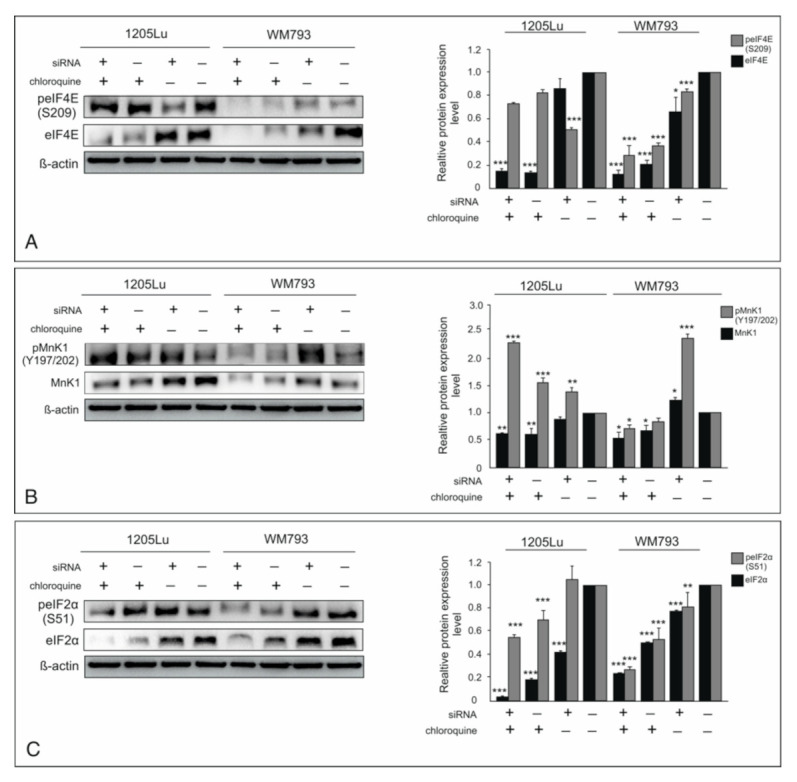
The inhibitory effect of ILK knock-down and CQ use on translation melanoma cells. “+” refers to siRNA ILK or chloroquine; “−” refers to silencer negative control cells. The expression of both phosphorylated and total proteins was determined by Western blotting and representative blots are displayed. The histograms are a quantitative representation of data after densitometry (mean ± SD) of three independent experiments. β-actin was used as a protein loading control. Asterisks indicate significant differences from control cells. Values are denoted as * *p* < 0.05, ** *p* < 0.01, and *** *p* < 0.001. (**A**)—eukaryotic translation initiation factor 4F (eIF4E), (**B**)—MNK1 kinase, (**C**)—eukaryotic initiation factor eIF2α.

Cancer cells require elevated protein synthesis as compared to their normal counterparts. Tumorigenesis is highly influenced by regulation of the cap-dependent translation, which needs the eukaryotic translation initiation factor 4F complex. A very important part of this complex is eIF4E [34]. We detected quite a pronounced expression level of eIF4E in both the melanoma cell lines. Both CQ and siRNA for ILK, whether they were used solo or in tandem, decreased the basal level of protein eIF4E significantly, however, most effective in both lines was their combination (Figure 5A). Phosphorylation of eIF4E on S209 is tightly regulated by MNK1/2 kinases and plays an important role in the translational activity of eIF4E [35,36]. In the WM793 cell line, the decrease in eIF4E expression is accompanied by a decrease in S209 phosphorylation (Figure 5A). Whereas a drop in phosphorylation on S209 was detected in all cases in 1205Lu, a very significant decrease was observed only after the application of ILK silencing. The maintenance of phosphorylation on S209 in 1205Lu seems to be a consequence of an increase in the activity of MNK1 kinase in the presence of CQ or silencing of ILK. A high level of phospho-Mnk 1 was also observed after ILK silencing in the WM793 cell line (Figure 5B).

Another key component of translational machinery is eukaryotic initiation factor eIF2, the role of which is to supply the initiator tRNA to the ribosome. Phosphorylation of the α subunit of eIF2 on S51 attenuated the general protein translation while facilitating the translation of selected proteins such as transcription factor ATF4 [37]. The use of either CQ or siRNA for ILK, as well as both of them simultaneously significantly reduced the basic level of the eIF2α subunit, and also lowered the level of phosphorylation but not as visibly as the total level of protein (Figure 5C).

### 2.7. Migration

**Figure 6:**
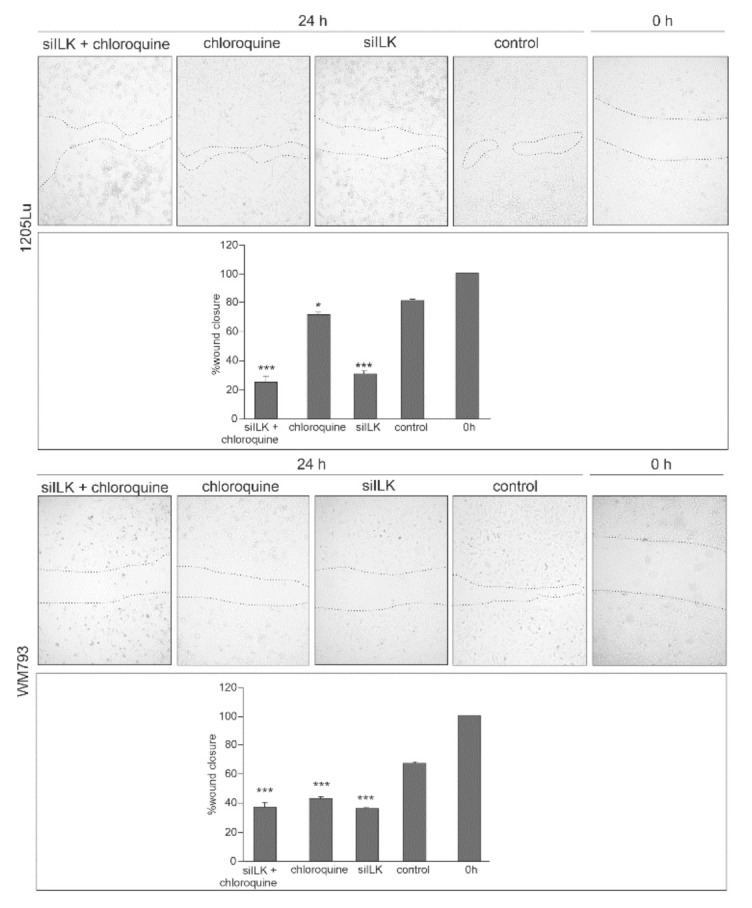
The effect of ILK knock-down and CQ use on in vitro wound healing/migration assay. After 24 h, the confluent cell monolayer was wounded and wound closure was captured by a digital camera connected to the inverted microscope. The assay was repeated thrice in duplicate. The area of wound closure was measured with the use of ImageJ and expressed as the percentage of wound closure. Asterisks indicate significant differences from control cells. The histograms are a quantitative representation of data (mean ± SD) of three independent experiments. Values are denoted as * *p* < 0.05, ** *p* < 0.01, and *** *p* < 0.001.

To evaluate the effects of CQ and knockdown of ILK on melanoma cell migration, we performed a scratch wound healing assay. In our previous work, we showed that ILK silencing reduces the migration of melanoma cells in the Boyden chamber [19] and also decreases transendothelial cell migration [21]. We adopted an in vitro wound healing/migration assay for evaluation of the potential enhancement of the anti-cancer effect of siILK by using it together with CQ. The results show that silencing of ILK significantly reduced (by about 60%) wound closure after 24 h in both melanoma cell lines. We observed a slight supporting effect of CQ treatment on reduction of migration in 1205Lu but a significant effect in WM793 (58%). In the case of combination treatment with CQ and siILK, the migration reduction effect is maintained at the level of siRNA for ILK treatment results (Figure 6).

## 3. Discussion

In our study, we demonstrated that silencing of ILK induced autophagy much more frequently in the early stage of melanoma compared to metastatic, and it may lead independently to cell death or act as a precursor of apoptosis. Our previous studies have revealed the important role of ILK in melanoma EMT and particularly in the regulation of N-cadherin expression by modulation of the expression of Rab proteins associated with N-cadherin endocytosis, degradation, or recycling. The observed increase in Rab9 expression after ILK silencing suggested that ILK may be in part responsible for the regulation of autophagy [21]. The overexpression of ILK inhibits autophagy by activation of Akt and mTOR [38]. On the other hand, autophagic disruption of E-cadherin/β-catenin interaction promotes EMT by upregulation of ILK [39]. It was reported that EMT-connected signaling pathways influence autophagy. The complicated link between autophagy and EMT seems to be a good target for cancer treatment [40]. Inhibition of endogenous autophagy by CQ is related to decreased cell survival in both herein-studied melanoma cell lines. These results remain in agreement with data showing that CQ is effective in growth inhibition in a variety of cancers, including melanoma, oral squamous cell carcinoma, or bladder cancer [11,12,16]. Apart from autophagy inhibition, the anti-cancer profile of chloroquine also involves mechanisms such as inhibition of proliferation, induction of apoptosis, or influencing tumor vasculature [11,12,13,14].

The crucial reason for the decrease of melanoma cells survival is probably the decline in the rate of translation by the decrease in expression of eukaryotic translation initiation factors such as eIF4E and eIF2α, observed after silencing of ILK and CQ treatment as well as the simultaneous use of both. Cancer cells require elevated protein synthesis, especially proteins necessary for an invasive phenotype. Both global protein synthesis and the selective translation of oncogene mRNAs are necessary for tumor growth, proliferation, and survival [34]. The crosstalk between translation and autophagy is disturbed in cancer [41]. The translation of tumor-associated proteins is controlled by mitogen-activated translational factor eIF4E, the expression of which is elevated in cancer cells [42]. Its availability and activity are controlled by mTORC1 through regulation of eIF4E inhibitor (4E-BP1) and MNK1/2 kinase, which phosphorylates eIF4E on S209. Phosphorylated eIF4E (peIF4E) preferentially enhances expression of oncogenic proteins [35]. We observed a decreased expression of eIF4E as well as a decreased phosphorylation on S209 in all cases of treatment; however, in metastatic cell lines 1205Lu after CQ treatment, the decrease in phosphorylation is slight but accompanied by an amazingly low protein level. BRAF mutation in melanoma cells results in constitutive activation of the MEK/ERK pathway and activation of MNK1/2 [43]. A major mechanism for global translational control involves phosphorylation of the α subunit of eIF2 on S51, which represses the delivery of initiator methionyl-tRNA to the translational machinery [34]. Paradoxically, eIF2α phosphorylation enhances the translation of selected mRNA, in particular transcription factor ATF4, which activates the autophagy genes LC3B and ATG5 [44]. In the absence of eIF2α phosphorylation, despite the inhibition of mTORC1, induction of autophagy is not possible in amino acid deprivation conditions [45]. This is evidence for eIF2 phosphorylation being necessary for autophagy induction. Despite the observed decrease in eIF2 α expression, the phosphorylation at S51 is kept at a relatively high level. Wengrod and colleges have shown that mTORC1 regulation of autophagy in melanoma needs eIF2α phosphorylation in a pathway independent of ULK1/2 [45].

**Figure 7:**
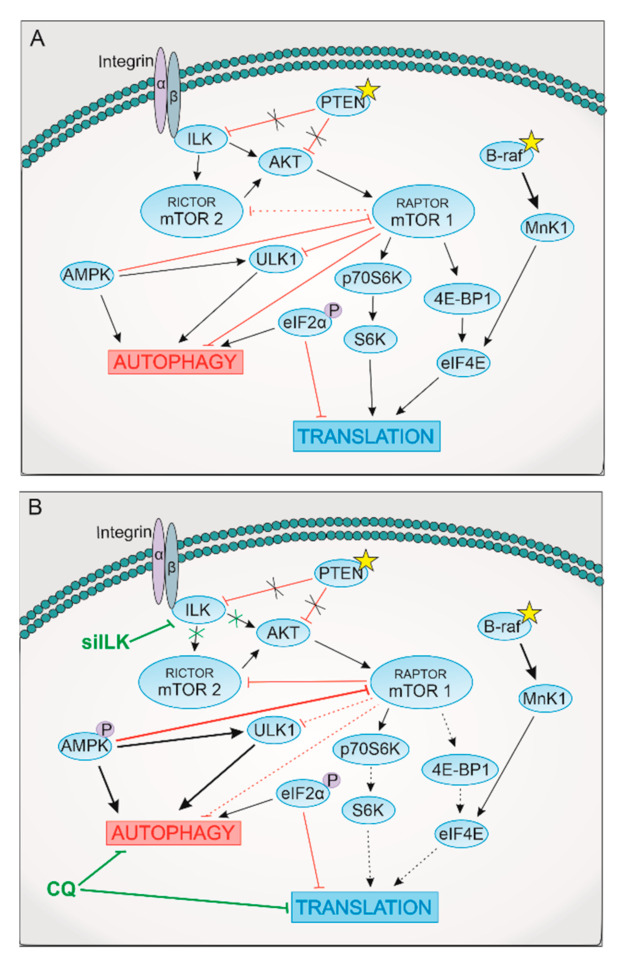
Model of the signaling pathway in tested melanoma cells. Red color—inhibition signals, black color—stimulation signals. (**A**) Mechanism of the ILK regulation of translation and autophagy in melanoma cells. (**B**) The influence of siILK and CQ treatment melanoma signaling.

ULK1 plays a secondary role in autophagy; monitoring changes in the activity of ULK1 is not a direct assay for autophagy activity. Additionally, ULK1 is phosphorylated by multiple kinases, and the number of phosphorylation sites can increase or decrease during autophagy induction [30]. However, the observed decrease in the phosphorylation status of ULK1 at inactivating sites (S757) in both tested melanoma cell lines in all treatments indicates the existence of autophagy-promoting conditions. The observation of downstream targets of mTORC1 did not show a consistent variation in its protein level or phosphorylation status in either of the cell lines. siRNA for ILK or CQ may likely affect the integrity of mTORC1, and a decreased or increased phosphorylation status of one of the TORC1 substrates does not necessarily correlate with changes in others. A decrease of mTORC1 activity is a good measure for translation inhibition and autophagy induction; however, autophagy may be induced by mTOR-independent mechanisms. Moreover, autophagy induction can cause negative feedback that results in the reactivation of mTOR. Both RAPTOR and RICTOR may influence autophagy via mTOR. A marked increase in both protein levels in metastatic melanoma cell 1205Lu is accompanied by lower autophagy induction after silencing of ILK, or by activation of pro-survival signals by an increase of Akt phosphorylation resulting from CQ treatment. Furthermore, the ILK/RICTOR complex is known to phosphorylate Akt and induce EMT [23,46]. RICTOR is speculated to be involved in the development of drug resistance in tyrosine kinase inhibitors therapy [47]. Perhaps the mTORC2 activation observed as an increase in S2481 phosphorylation is the effect of RICTOR’s release from the ILK complex after silencing in 1205Lu cells, or the activation of mTORC2 is a feedback mechanism to enhance cell survival after CQ treatment. The increase in mTORC2 activity may be the reason for the weak influence of CQ on the inhibition of migration of 1205 Lu cells. Combinatorial treatment of siRNA for ILK and CQ causes both mTORC1 and mTORC2 inhibition monitored as phosphorylation on S2448 and S2481 (Figure 7).

Because ILK is at the heart of the interplay between translation and autophagy, targeting the autophagy machinery by combinatorial treatment with autophagy activator (siRNA for ILK) and autophagy inhibitor CQ causes autophagy dysregulation with positive anticancer effects. The presented results demonstrated that such an approach produces a stronger antitumor effect compared to each treatment used alone. An especially significant decrease in melanoma cells proliferation was observed. However, the underlying mechanism against tumor is ambiguous and does not appear to be dependent on leading tumor mutations. Our findings show a complex interaction between signaling pathways and activation of alternative compensatory pathways promoting survival triggered by autophagy induction or inhibition. Autophagy regulation remains intriguing as a potential stand-alone therapeutic strategy or as an enhancer of the sensitivity of cancer cells to other conventional drugs.

## 4. Materials and Methods

### 4.1. Cell Culture

Human melanoma cell lines: WM793 (vertical growth phase—VGP) and 1205Lu (metastatic) cell line was derived from lung metastases of WM793 after subcutaneous injection into immunodeficient mice. 1205Lu cells are highly invasive and exhibit spontaneous metastases to the lung and liver. The BRAFVV600E and loss of PTEN coexist in both cell lines. Cells were grown in RPMI-1640 medium supplemented with 10% fetal bovine serum and penicillin/streptomycin (Gibco, Grand Island, NY, USA). Cells were obtained from the ESTDAB Melanoma Cell Bank (Tubingen, Germany). All cell lines are tested for mycoplasma infection by PCR.

### 4.2. Cell Culture Treatment

Melanoma cells were grown until 60–70% confluency was reached, and then transfected using INTERFERin™ as per the manufacturer’s protocol (Polyplus Transfection, Illkirch, France) with three different 21bp double-stranded siRNA molecules specifically targeting the ILK (Ambion ID#288570; ID#145116; ID#145117) or a control non-silencing sequence (Ambion ID#4611) (Thermo Fisher Scientific, Waltham, MA, USA).

For siRNA transfection and scratch wound healing assays, serum-free medium Opti-MEM (Gibco, Grand Island, NY, USA) was used. WM793 cells and 1205Lu were in each case transfected with 60 nM and 80 nM siRNA, respectively. As transfection control, melanoma cells were transfected 19 bp scrambled sequence with 3′dT overhangs. The sequences have no significant homology to any known human gene sequences. Because no differences were observed between un-transfected cells and silencer negative control cells, the latter were selected as control cells in other experiments. After 24 h, the medium was replaced with a fresh one and cells were grown for an additional 24 h period with or without chloroquine (Sigma-Aldrich, St. Louis, MO, USA) in the final concentration of 50 µM (48 h post-transfection) before analysis.

### 4.3. Western Blot Analysis

Cells lysis, Western blot, detection, and visualization of blots were carried out as we previously described [21]. Table 1 lists the antibodies and conditions that were used in this study. Cell Signaling Technology Inc., Danvers, MA, USA; Transduction Laboratories, BD Franklin Lakes, NJ, USA; Sigma-Aldrich, St. Louis, MO, USA; Calbiochem, Merck Darmstadt, Germany; MERCK Millipore, Darmstadt, Germany; Santa Cruz Biotechnology, Inc., Heidelberg, Germany.

### 4.4. Monitoring Autophagy in the Cell

Autophagy was monitored by sodium sulfate-polyacrylamide gel electrophoresis (SDS-PAGE) and Western blotting with LC3A/B antisera. Accumulation of LC3-II, which is associated with the number of autophagosome formation, was used as a good marker for monitoring autophagy progression. Because that LC3-II is both induced and degraded during autophagy, we observed autophagic flux also in the presence of chloroquine (CQ) (Sigma-Aldrich, St. Louis, MO, USA), an autophagy inhibitor that prevents the degradation of LC3-II and blocks endogenous autophagi flux.

### 4.5. Proliferation

The proliferation of cells was assessed with the crystal violet test, as previously described [48].

### 4.6. In Vitro Wound Healing/Migration Assay

The in vitro model of wound healing was used to compare the migration of melanoma cell lines after transfection of siRNA for ILK and in the presence or absence of CQ. For wound healing assay, cells were grown after transfection in serum-free medium Opti-MEM until confluent in 24 well plates (24 h). A small linear scratch was created in the confluent monolayer by gently scraping with a sterile 1 mL pipette tip. The cells were washed with medium to remove cellular debris before treating serum-free Opti-MEM media with or without chloroquine in concentration 50 µM. 24 h later, images of the migrated cells were taken using a digital camera (Nikon, Tokyo, Japan) connected to the inverted microscope. The assay will be repeated thrice in duplicate.

### 4.7. Statistical Analysis

Shapiro–Wilk W-test was used to check the normality of each variable. Levene’s test was used for the assessed homogeneity of variance. Statistical analyses of data from in vitro studies were performed by one-way analysis of variance (ANOVA) followed by Dunnett’s post hoc comparison test to determine which values differed significantly from the controls. All analyses were made using Statistica 13 software (StatSoft Inc., Tulsa, OK, USA). Data were presented as mean ± SD and considered statistically significant at * *p* < 0.05, ** *p* < 0.01, and *** *p* < 0.001.

## Figures and Tables

**Table 1:** Details of primary antibodies used for Western blot analysis.

Primary Antibody	Host Species	Dilution (Application)	Vendor
ILK	mouse	1:1000	BD Transduction Laboratories
PARP1	mouse	1:1000	Merck
LC3A/B	rabbit	1:1000	Cell Signaling Technology
AMPKα	rabbit	1:1000	Cell Signaling Technology
AMPKα Y172	rabbit	1:1000	Cell Signaling Technology
ULK1	rabbit	1:1000	Cell Signaling Technology
ULK1 S555	rabbit	1:1000	Cell Signaling Technology
ULK1 S757	rabbit	1:1000	Cell Signaling Technology
RICTOR	rabbit	1:1000	Cell Signaling Technology
RAPTOR	rabbit	1:1000	Cell Signaling Technology
mTOR S2448	rabbit	1:1000	Cell Signaling Technology
mTOR S2481	rabbit	1:1000	Cell Signaling Technology
β-actin	mouse	1:12000	Sigma Aldrich
mTOR	rabbit	1:1000	Cell Signaling Technology
70S6K Y389	rabbit	1:1000	Cell Signaling Technology
70S6K	rabbit	1:1000	Santa Cruz Biotechnology
S6	rabbit	1:1000	Santa Cruz Biotechnology
pS6 S235/236	rabbit	1:1000	Cell Signaling Technology
p4E-BP1	rabbit	1:1000	Cell Signaling Technology
Akt S473	rabbit	1:1000	Cell Signaling Technology
Akt	mouse	1:500	BD Transduction Laboratories
eIF4E S209	rabbit	1:1000	Cell Signaling Technology
eIF4E	rabbit	1:1000	Santa Cruz Biotechnology
pMnK1	rabbit	1:1000	Cell Signaling Technology
MnK1	rabbit	1:1000	Cell Signaling Technology
eIF2α	rabbit	1:1000	Santa Cruz Biotechnology
eIF2α S51	rabbit	1:1000	Cell Signaling Technology

## Data Availability

Data available on request from corresponding author.
